# Scientific advances in post-exercise hypotension: a bibliometric review

**DOI:** 10.1038/s41371-026-01129-6

**Published:** 2026-03-24

**Authors:** Samara Sezana-Costa, Eduardo Amadeu Dutra Moresi, Thiago dos Santos Rosa, Clarcson Placido Conceição Santos, Fernando Ribeiro, Alexandre Sergio Silva, Herbert Gustavo Simões, Marcos Doederlein Polito, Paulo Farinatti, Linda S. Pescatello, Hugo de Luca Correa, Milton Rocha Moraes

**Affiliations:** 1 Graduate Program in Physical Education Catholic University of Brasilia, Brasilia, Brazil; 2 Project Manager - Apple Developer Academy/Catholic University of Brasilia, Brasilia, Brazil; 3 Department of Physical Education, Graduate Program in Human Movement Sciences, State University of Northern Paraná, Jacarezinho, Brazil; 4 Graduate Program in Genomic Science and Biotechnology, Catholic University of Brasília, Brasilia, DF Brazil; 5 Research Group on Metabolic Diseases, Physical Exercise and Health Technologies, Bahiana School of Medicine and Public Health, Salvador, Brazil; 6 Institute of Biomedicine (iBiMED), School of Health Sciences, University of Aveiro, Aveiro, Portugal; 7 Department of Physical Education, Laboratory of Physical Training Studies Applied to Performance and Health, Federal University of Paraíba, João Pessoa, Brazil; 8 Research Group of Cardiovascular Response and Exercise, Londrina State University, Londrina, Paraná, PR Brazil; 9 Laboratory of Physical Activity and Health Promotion, Institute of Physical Education and Sports, University of Rio de Janeiro State, Rio de Janeiro, RJ, Brazil;, University of Rio de Janeiro State, Rio de Janeiro, Brazil; 10 Department of Kinesiology, University of Connecticut, Storrs, CT USA; 11 Department of Physical Education, College of Education and Technology Phorte, São Paulo, Brazil

**Keywords:** Hypertension, Cardiovascular diseases

## Abstract

This study aimed to perform a bibliometric review to map scientific advances and research trends related to post-exercise hypotension (PEH), a transient blood pressure reduction following physical exercise, increasingly recognized as a non-pharmacological strategy for hypertension management. A comprehensive search was conducted on Scopusdatabase to identify publications on PEH from 1985 to 2024. Metadata from 440 selected articles were analyzed using VOSviewer™ and Gephi™ software to construct keyword co-occurrence networks and identify emerging research topics, influential authors, and leading institutions. Bibliometric analysis revealed an increasing trend in PEH publications over the past decade, with Brazil and the United States leading in research output. High-intensity interval training, isometric resistance exercise and nutritional interventions emerged as key topics. Findings emphasized exercise intensity, recovery posture, and dietary strategies modulate PEH magnitude and duration. Complex autonomic and vascular factors were consistently implicated as physiological mechanisms of PEH, supporting the importance of individualized exercise prescriptions. This review highlights the growing clinical and scientific importance of PEH research. Notable gaps include the need for standardized assessment methodologies, greater biomarker utilization, and further evaluation of multimodal exercise–nutrition strategies to improve clinical translation. Future studies should expand PEH research in clinically high-risk and underrepresented groups (e.g., older adults and individuals with cardiometabolic or renal comorbidities), using harmonized protocols to enhance comparability and applicability.

## Introduction

Post-exercise hypotension (PEH) consists of a transient and clinically significant reduction in blood pressure (BP) following a single exercise session compared to a non-exercise control condition [1–3]. BP reductions areon average 5-8 mmHg lasting up to 12–72 h post-exercise [4–7].

Initially observed by Hill in 1897 following a 400-yard run [8], PEH is currently recognized as a multifactorial phenomenon involving complex central and peripheral mechanisms, such as nitric oxide-mediated vasodilation, reductions in peripheral vascular resistance, and autonomic nervous system modulation [4, 9]. Despite significant advancements in understanding these mechanisms, reports on the magnitude and duration of PEH remain inconsistent, primarily due to methodological heterogeneity and the lack of standardized protocols for hemodynamic assessment [6, 10, 11].

Continuous aerobic exercise is widely recommended to elicit PEH, particularly in sedentary individuals with elevated BP [4, 12]. Alternative approaches such as high-intensity interval training (HIIT) [9], dynamic resistance exercise [2, 6], and isometric exercise modalities [13–16] have also shown promise, though modality comparisons are challenged by methodological differences.

Multiple factors, including exercise intensity, recovery posture, and nutritional interventions, modulate the PEH response [5, 17, 18]. Nutritional supplementation with inorganic nitrate or L-arginine may enhance PEH [17, 19], while megadoses of vitamin D mayattenuate the response [20]. Methodological variations, such as differences in exercise protocols, timing of BP measurements, and settings (laboratory vs. ambulatory), as well as individual characteristics like age, sex, and ethnicity contribute to the variability in PEH outcomes [12, 21]. Additionally, biomarkers such as histamines [11], 15-HETE [22], and ATP [23] have been implicated in modulating PEH.

Although the acute BP-lowering effects of PEH are well established, the impact of chronic exercise training on the magnitude and duration of PEH remains unclear, with existing literature presenting conflicting findings, particularly regarding adaptations such as reductions in resting BP [2, 9, 24]. In light of these uncertainties, a bibliometric review offers a valuable approach to consolidating existing knowledge, mapping research trends, and uncovering areas in need of further investigation [25]. Accordingly, the objective of this reviewwas to conduct a bibliometric analysis of PEH-related research to synthesize key scientific developments and highlight current challenges within the field.

## Methods

A bibliometric review on PEH was conducted by systematically searching the Scopus database using the keyword “post-exercise hypotension.” Scopus was included in our search strategy to ensure comprehensive coverage of peer-reviewed studies in exercise physiology and cardiovascular health.The search covered publications from 1985 to 2024 and initially yielded 493 documents. To improve methodological rigor, filters were applied to include only journal articles, reviews, and conference papers published in English, resulting in a final dataset of 440 records.

Metadata from the selected studies were extracted and imported into VOSviewer™ software, to generate a co-occurrence network of author keywords that appeared at least twice across the dataset. The initial network consisted of 183 nodes distributed into 16 communities, interconnected by 1027 edges [26–29].

Vocabulary standardization was performed using the Thesaurus function in VOSviewer™ to address ambiguities, synonyms, and plural forms, resulting in a refined co-occurrence network comprising 139 nodes distributed across five communities, with a total of 826 edges. The processed metadata were subsequently exported in CSV format and analyzed using bibliometric methods as outlined by Moresi, Pinho, and Costa, 2021 [28]. To complement the network analysis, additional visualizations and network metrics, including average degree, modularity, and clustering coefficientwere computed using Gephi™ software [27].

## Results

A total of 440 articles related to PEH were identified and subjected to bibliometric analysis. The results are presented in terms of publication trends, institutional contributions, keyword co-occurrences, authorship networks, geographic distribution, and journal rankings.

**Fig. 1:**
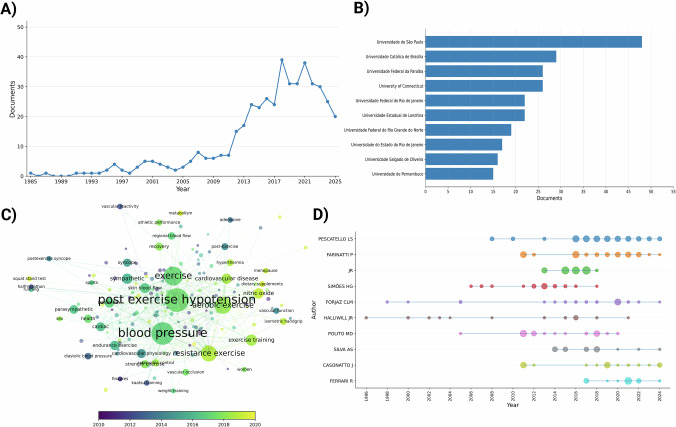
Comprehensive summary of documents related to post-exercise hypotension. **A** Post-exercise hypotension published articles by year. **B** Post-exercise hypotension published articles by university affiliation of origin. **C** Thermal density of keywords related to post-exercise hypotension. **D** The authors’ publication frequency of post-exercise hypotension articles from 1996 to the present. Authors: Pescatello LS (Linda Shannon Pescatello); Farinatti P (Paulo Farinatti); MacDonald JR (Jay R.MacDonald); Simões HG (Herbert Gustavo Simões); Forjaz CLM (Claudia Lucia Moraes Forjaz); Halliwill JR (Jonh R. Halliwill); Polito M (Marcos DoederleinPolito); Silva AS (Alexandre Sérgio Silva); Cassonato J (Juliano Cassonato); Ferrari R (Rodrigo Ferreira Ferrari).

The number of PEH-related publications remained relatively stable between 1985 and 2010, with fewer than 10 articles published annually during this period. A steady increase was observed thereafter, reaching a peak of 39 publications in 2017 (Fig. 1A). A decrease in publication rates occurred after 2020, possibly reflecting the effects that global events had on overall research output.Institutional analysis showed Brazilian universities were the most prolific contributors, with the University of São Paulo (USP) leading with 48 publications (Fig. 1B). Among the top 10 institutions, five were located in Brazil, highlighting the country’s substantial engagement in PEH research.

The keyword co-occurrence analysis revealed “post-exercise hypotension” as the most frequent term, appearing in 217 articles, followed by “blood pressure” (122 articles), “exercise” (97 articles), “aerobic exercise” (85 articles), and “resistance exercise” (78 articles) (Fig. 1C). Table 1 lists the keywords with the highest eigenvector centrality values.

**Table 1:** Keywords Related to Post-Exercise Hypotention with the Greatest Influence on Eigenvector Centrality.

1	BloodPressure
2	Post ExerciseHypotension
3	Exercise
4	Hypertension
5	ResistanceExercise
6	Heart Rate
7	Hypotension
8	AerobicExercise
9	AmbulatoryBloodPressureMonitoring
10	PhysicalActivity
11	Cardiac
12	AutonomicNervous System
13	Hemodynamic
14	Baroreflex
15	Endurance Exercise
16	Sympathetic
17	Aging
18	Health
19	Elderly
20	Nitric Oxide

Authorship analysis demonstrated a consistent increase in collaborative publications over time, with prominent contributors including Pescatello LS, Farinatti P, MacDonald JR, and Simões HG (Fig. 1D). The average number of authors per publication was 6.15, indicating a strong trend toward collaborative research in this field.

**Fig. 2:**
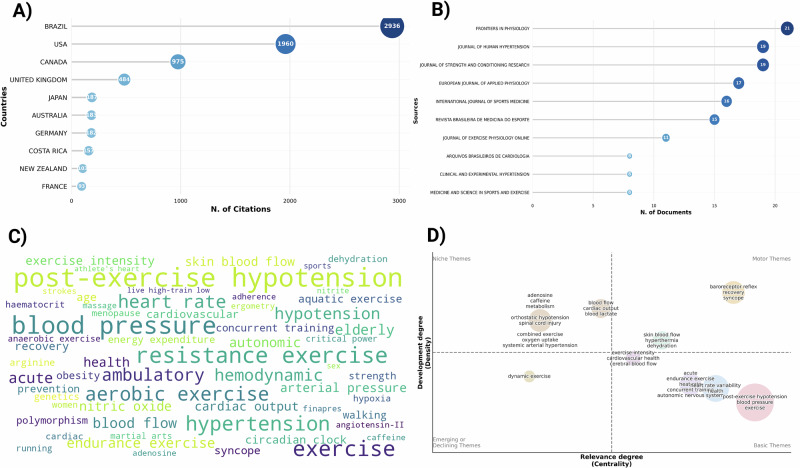
Scientific landscape of post-exercise hypotension research. **A** Number of post-exercise hypotension citations by country of origin. **B** Top 10 journals regarding the number of published post-exercise hypotensionarticles. **C** Cloud of keywords in the post-exercise hypotension literature. **D** Thematic mapcategorizing of post-exercise hypotension topics.

Country distribution analysis indicated that Brazil accumulated the highest number of citations (1323), followed by the United States (416) and Canada (129) (Table 2, Fig. 2A). (Table 2, Fig. 2A).

**Table 2:** Number of post-exercise hypotension published articles by country of origin.

Country	Publication Frequency
Brazil	1323
USA	416
Canada	129
UK	124
Japan	122
Italy	59
Australia	49
Portugal	48
Germany	43
Iran	40

The journals publishing the greatest number of PEH-related articles were *Frontiers in Physiology*, *Journal of Human Hypertension*, and *Journal of Strength and Conditioning Research* (Fig. 2B). The impact factors of the top 10 journals ranged from 0.4 to 6.3 (Table 3).

**Table 3:** Top 10 journals publishing post-exercise hypotension articles, ranked from highest to lowest impact factor.

Ranking	Journal	IF
1st	Medicine and Science in Sports and Exercise	6.3
2nd	FrontiersPhysiology	4.0
3rd	JournalofHumanHypertension	3.0
4th	European Journal of Applied Physsiology	2.6
5th	Clinical and Experimental Hypertension	2.5
6th	International Journal of Sports Medicine	2.4
7th	Journal of Strength And Conditioning Research	2.1
8th	Brazilian Archives of Cadiology	1.1
9th	Brazilian Journal of Sports Medicine	0.4
10th	Journal of Exercise Physiology Online	N.A.

Journal Citation Reports (JCR/Clarivate); *IF* impact factor, *N.A*. not available.

A keyword cloud visualization (Fig. 2C) highlighted the dominance of terms such as “blood pressure,” “exercise,” and “vascular function” across the analyzed literature.Thematic mapping of the bibliometric network (Fig. 2D) identified four major research clusters; 1^st^ (post-exercise hypotension, blood pressure, exercise); 2^nd^ (heart rate variability, autonomic nervous system, health); 3^rd^ (baroreceptor reflex, recovery, synce); 4^th^ (acute, endurance exercise, concurrent training) based on density and centrality, providing insight into the structure and development of PEH-related research themes.

## Discussion

This bibliometric review offers a comprehensive overview of the scientific landscape of PEH research from 1985 to 2024. The analysis revealed a marked increase in PEH-related publications over the past decade, with Brazilian institutionsemerging as prominent contributors to the field. The keyword co-occurrence network highlighted exercise modality, blood pressure regulation, and vascular function as central thematic areas, underscoring their pivotal roles in shaping the direction of PEH research.Brazil’s prominent contribution likely reflects long-standing, highly productive research groups in exercise physiology and blood pressure regulation. However, this concentration also highlights the need for broader multinational, multi-center collaboration to improve generalizability across healthcare systems and patient profiles.

The growing number of studies focusing on alternative exercise modalities, such as HIIT and isometric resistance exercise, reflects an evolving understanding of PEH beyond traditional aerobic paradigms. These findings are consistent with previous reviews suggesting exercise intensity, volume, and recovery posture significantly modulate the PEH response [5, 6, 24]. Furthermore, the observed emergence of nutritional interventions in the keyword network underscores the increasing interest in combining exercise with dietary strategies to optimize BP control.

To support comparability and clinical translation, we propose a minimum methodological reporting framework for PEH studies: (i) standardized baseline BP acquisition ( ≥ 5–10 min seated rest; ≥2–3 measurements; device and cuff specification); (ii) explicit exercise prescription reporting (modality, duration, and intensity definition anchored to clinically interpretable domains, e.g., %HRR, %HRpeak, %VO₂peak/ventilatory thresholds or RPE); (iii) standardized recovery conditions (posture, ambient temperature, hydration/caffeine/meal timing, and medication timing when applicable); (iv) harmonized post-exercise BP timepoints (early recovery and later timepoints) and, when feasible, ambulatory BP monitoring to capture duration; and (v) clear PEH definition relative to a non-exercise control condition and transparent handling of inter-individual variability (e.g., responder analyses).

From a clinical perspective, PEH assessment can be operationalized as a pragmatic monitoring tool within supervised exercise programs. For example, clinicians may measure seated BP after standardized rest before exercise and again during standardized recovery (e.g., at 10–15 min and 30–60 min post-session under controlled posture), using the individual’s PEH profile (magnitude and persistence) to inform exercise prescription refinement and safety monitoring. Such an approach should be interpreted alongside symptoms, medication timing, and ambulatory BP patterns when available, and it complements (rather than replaces) established hypertension management strategies.

Notably, bibliometric data revealed a strong collaborative network among leading authors and institutions, suggesting the field benefits from multi-center and interdisciplinary research efforts. However, the analysis also highlighted substantial methodological heterogeneity across studies, including differences in BP measurement timing, exercise prescription protocols, and participant characteristics. These inconsistencies continue to limit the comparability of PEH studies and underline the urgent need for standardized assessment methodologies, as previously recommended [1, 11, 12].

This review has several strengths, including the systematic and quantitative mapping of research outputs, the application of advanced bibliometric techniques, and the integration of multiple bibliographic tools (VOSviewer and Gephi). However, certain limitations must be acknowledged. The exclusive use of a single search term (‘post-exercise hypotension’) may have led to the omission of relevant studies employing alternative terminology. Furthermore, the restriction to English-language publications may have introduced a language bias, potentially limiting the comprehensiveness of the analysis.We emphasize that these populations are proposed as clinically relevant examples rather than as priorities derived directly from bibliometric mapping. This rationale reflects the high prevalence and clinical burden of hypertension and vascular dysfunction in older adults and in cardiometabolic/renal disease, alongside the need for more subgroup-specific PEH studies using standardized methods to support clinical translation.

Future research should focus on addressing identified gaps, including the integration of biomarkers to elucidate the physiological mechanisms underlying PEH variability. Greater emphasis on long-term adaptations to exercise training and standardized methodologies is critical to enhance the clinical applicability of PEH findings. Multimodal strategies combining exercise with nutritional supplementation, particularly in high-risk populations such as older adults, individuals with renal disease, and those with metabolic disorders, represent promising avenues for further exploration.

In conclusion, this bibliometric review highlights the dynamic growth of PEH research over the past four decades, identifies key trends and contributors, and underscores critical methodological gaps. These findings provide a strategic foundation for advancing the field toward more standardized, mechanistic, and clinically relevant investigations.
